# 

**Published:** 1849-01

**Authors:** 


### THE
### WESTERN JOURNAL
### OF
### MEDICINE AND SURGERY:

EDITED BY

LUNSFORD P. YANDELL, M. D.,

PROFESSOR OF CHEMISTRY AND PHARMACY IN THE UNIVERSITY OF LOUISVILLE.

NO. CIX.—JANUARY, 1849.

**THIRD SERIES.**

Vol. III.....No. I.

LOUISVILLE, KY:

PUBLISHED MONTHLY BY PRENTICE AND WEISSINGER,

PROPRIETORS AND PRINTERS.

1849.

